# Improving risk-stratification of rheumatoid arthritis patients for interstitial lung disease

**DOI:** 10.1371/journal.pone.0232978

**Published:** 2020-05-08

**Authors:** Jérôme Avouac, Anne Cauvet, Alexia Steelandt, Yuichiro Shirai, Muriel Elhai, Masataka Kuwana, Oliver Distler, Yannick Allanore

**Affiliations:** 1 INSERM U1016 and CNRS UMR8104, Cochin Institute, Paris University, Sorbonne Paris Cité, Paris, France; 2 Rheumatology department, Cochin Hospital, Paris University, Sorbonne Paris Cité, Paris, France; 3 Allergy and Rheumatology, Nippon Medical School Graduate School of Medicine, Tokyo, Japan; 4 Department of Rheumatology, Zurich, Switzerland; Medical Center - University of Freiburg, GERMANY

## Abstract

**Objective:**

To determine the performance of 3 circulating markers for the diagnosis and the progression of interstitial lung disease (ILD) associated with rheumatoid arthritis (RA).

**Methods:**

Serum concentrations of 3 circulating markers, lung epithelial-derived surfactant protein D (SPD), chemokine CCL-18 and Krebs von den Lungen-6 glycoprotein (KL-6), were measured by ELISA in consecutive patients with established RA. These patients were recruited from 3 tertiary centers and they all had been investigated by chest high-resolution computed tomography (HRCT). For a subset of French patients, a follow-up HRCT was available (mean interval between HRCT: 3±1.5 years).

**Results:**

Among the 147 included patients (age: 66 ± 12 years, 69% women, disease duration 11 ± 10 years), 40 (27%) had RA-ILD on chest HRCT. SPD, CCL18 and KL-6 concentrations were significantly higher in patients with RA-ILD. ROC curve analysis to assess the diagnostic abilities of the three markers for the diagnosis of RA-ILD showed a superiority of KL-6 (Area under the curve, AUC: 0.79 95% CI 0.72–0.86) compared to SPD (AUC: 0.66 95% CI 0.58–0.74) and CCL18 (AUC: 0.62, 95% CI 0.53–0.70). The sensitivity of KL-6 for the diagnosis of RA-ILD was 68% with a specificity of 83%. The combination of KL-6 with SPD and CCL18 improved its diagnostic ability, with increased sensitivity from 68% to 77%, specificity from 83% to 97%. Increased KL-6 levels were independently associated with the presence of RA-ILD after the adjustment on other RA-ILD risk factors. In the French subset with longitudinal data, baseline KL-6 serum levels were predictive of ILD progression and the degree of ILD progression on HRCT was proportional to baseline KL-6 concentrations.

**Conclusion:**

These results show that KL-6 is a relevant circulating marker for the diagnosis and might be an interesting marker for the progression of RA-ILD.

## Introduction

Interstitial lung disease (ILD) is the most common pulmonary manifestation of rheumatoid arthritis (RA), occurring in ∼10% of patients. It has emerged in recent series as a key prognostic factor including survival [1]. RA-ILD shares some genetic and phenotypic similarities with other fibrotic diseases including idiopathic pulmonary fibrosis, supporting the use of the same drugs in these conditions [2, 3]. Of great interest, the INBUILD trial recruited a broad range of progressive fibrosing ILD, including patients with RA; it showed that RA patients who received nintedanib had a slower rate of progression of ILD than those who received placebo [3]. Nevertheless, the big challenge for rheumatologists is now the risk-stratification of RA patients for ILD. Chest high-resolution computed tomography (HRCT) is the gold standard for RA-ILD diagnosis, but costs and ionizing radiation may limit its use in clinical practice. Thus, circulating biomarkers could aid in this risk-stratification, as recently reported in systemic sclerosis (SSc)-associated ILD [4–6]. Indeed, circulating lung epithelial-derived surfactant protein D (SPD), CCL-18 and Krebs von den Lungen-6 glycoprotein (KL-6) were identified as relevant diagnostic and prognostic markers of SSc-ILD. Our objective was to evaluate the merit of these 3 circulating markers for the diagnosis and the progression of RA-ILD.

## Patients and methods

### Inclusion criteria

We included consecutive patients with RA, >18 years of age, from 3 tertiary rheumatology centers (Paris, France, Tokyo, Japan and Zurich, Switzerland) over a 36-month period. All patients fulfilled the 1987 American College of Rheumatology (ACR) or the 2010 ACR/European League Against Rheumatism (EULAR) classification for RA. They were recruited because they had been investigated by routine chest HRCT performed during the inclusion period [7, 8]. All included patients agreed to participate in the study after informed consent, which was recorded in the medical source file. The protocol and the informed consent document have received Institutional Review Board/Independent Ethics Committee (IRB/IEC) approval before initiation of the study (“Comité de Protection des Personnes” Paris Ile de France I).

### Data collection from RA patients

History taking, physical examination, laboratory tests, and review of medical files were systematically performed to collect data from RA patients. Current / past medication use were obtained from information provided by patients, and based on the review of medical records. All patients had at least one chest HRCT and one measurement of forced vital capacity (FVC) and diffusing capacity of lung for carbon monoxide (DLCO) performed during the inclusion period. In the subset of French patients with ILD, HRCT lung images and pulmonary function tests (PFTs) were obtained both at baseline (time of blood sample collection) and at a follow-up visit. The ILD status of patients with RA was established by chest HRCT. The chest HRCT pattern was classified as usual interstitial pneumonia, UIP or non-specific interstitial pneumonia, NSIP [9]. Extent of fibrosis on baseline and follow-up HRCT was measured as previously described and expressed as the percentage of fibrosis on total lung volume [10]. In Paris, chest HRCT were examined directly on computer thanks to Picture Archiving and Communication Systems (PACS) and analyzed by two independent investigators blinded for the IDL status of RA patients. The intra- and inter-rater reliability (intraclass correlation coefficient, ICC) for the diagnosis of RA-ILD was 0.87 and 0.85, respectively. The intra- and inter-rater reliability (ICC) for the extension of RA-ILD was 0.75 and 0.70, respectively. In Tokyo, two independent investigators examined chest HRCT under a blinded manner. The intra- and inter-rater reliability (ICC) for the diagnosis of RA-ILD was 0.89 and 0.87, respectively. In Zurich, chest HRCT were examined by a single investigator blinded for the ILD status of RA patients. The intra-rater reliability (ICC) for the diagnosis of RA-ILD was 0.91. RA-ILD progression was defined in the subset of French patients with ILD by a progression >10% on HRCT and either a FVC decline >15% or a FVC decline >10% combined with a DLCO decline >15% [4, 11].

### Quantitative analyses of candidate serum biomarkers

Blood samples were obtained upon standardized procedures and processing [4]. Chest HRCT and FVC results were considered if performed within 6 months of each serum sampling. Blood was collected during a standard of care blood draw, then centrifuged at room- temperature within 30 minutes and serum aliquots were stored at -70°C until assayed. Circulating KL-6 was analyzed by ELISA kit (Sekisui Medical Co., Tokyo, Japan), SP-D by ELISA Kit ((DSFPD0)—Bio-techne, Rennes, France), CCL18 by ELISA Kit ((DCL180B)—Bio-techne). According to the manufacturer’s protocol, standard samples were run in duplicate and the coefficient of variation (Cv) were respectively less than 15%, 9.3% and 9.1%.

### Statistical analysis

Analyses were performed using MedCalc (v18.9.1). Categorical results were presented as counts and percentages, and continuous variables as means ± standard deviations (SD). Chi- squared test and Student’s t-test were used as appropriate. The ability of the biomarkers to diagnose RA-ILD was assessed by receiver operating characteristic (ROC) curve analysis. Sensibility, specificity, positive predictive value (PPV) and negative predictive value (NPV) were calculated. A multivariate analysis by logistic regression was also performed to determine the factors independently associated with the presence of RA-ILD. This analysis included RA-ILD as the dependent variable and RA-ILD risk factors as covariates.

## Results

### Study population

The mean age of the 147 included RA patients was 66 ± 12 years, 69% were women, and mean disease duration was 11 ± 10 years. Patients with RA-ILD (n = 40, age at onset: 56 ± 16 years) were more likely to be older than RA patient without ILD, with a higher proportion of males, smokers (past or current), and higher frequency of positive anti-citrullinated protein antibodies (ACPA). Although the use of conventional synthetic disease modifying anti-rheumatic drugs was similar between RA patients with or without ILD, RA-ILD patients were less likely to receive methotrexate (MTX) (**Table 1**). Detailed characteristics of patients are provided in **Table 1**. Among these patients, 40 (27%) had fibrosing ILD on HRCT (23 from Tokyo, 15 from Paris and 2 from Zurich): 21 had a UIP pattern, 17 a NSIP pattern, and 2 had NSIP associated with chronic obstructive pulmonary disease.

**Table 1:** Characteristics of included patients.

	Rheumatoid arthritis ILD- (n = 107)	Rheumatoid arthritis ILD+ (n = 40)	P-value
**Age (years), mean±SD**	**62±12**	**71±15**	**<0.001**
**Women, n (%)**	**80/107 (75)**	**22/40 (55)**	**0.019**
**Disease duration (Years), mean±SD**	**12±9.5**	**12±11**	**0.987**
**Past or current smokers, n (%)**	**28/107 (26)**	**24/40 (60)**	**<0.001**
**Erosions, n (%)**	**64/102 (63)**	**20/40 (50)**	**0.154**
**Positive ACPA antibodies, n (%)**	**79/104 (76)**	**38/40 (95)**	**<0.001**
**ACPA levels, mean, UI/L (range)**	**651 (0–4510)**	**245 (0–1564)**	**0.064**
**Positive rheumatoid factor, n (%)**	**77/104 (74)**	**34/40 (85)**	**0.160**
**Mean extension of ILD on HRCT (%), mean (range)**	**NA**		**NA**
**Mean FVC (%), mean (range)**	**NA**	**15 (5–50)**	**NA**
**Mean DLCO (%), mean (range)**	**NA**	**79 (28–138)**	**NA**
		**61 (20–114)**	
**Treatments**			
• **Corticosteroids, n (%)**	**73/100 (73)**		**0.065**
• **Conventional synthetic DMARDs, n (%)**	**76/107 (71)**	**32/37 (88)**	**0.637**
• **Methotrexate, n (%)**	**66/107 (62)**	**30/40 (75)**	**<0.001**
• **Targeted biological therapies, n (%)**	**14**	**12/40 (30)**	
**TNFα inhibitors, n**	**16**	**3**	
**Rituximab, n**	**8**	**9**	
**Tocilizumab, n**	**4**	**4**	
**Abatacept, n**		**11**	

SD: standard deviation, ACPA: anti-citrullinated protein antibodies ILD: interstitial lung disease, HRCT, high resolution computed tomography, DMARDs disease modifying anti-rheumatic drugs, FVC: forced vital capacity, DLCO: diffusing capacity of lung for carbon monoxide, NA: not applicable

### Performance of serum markers for the diagnosis of RA-ILD

**Fig 1:**
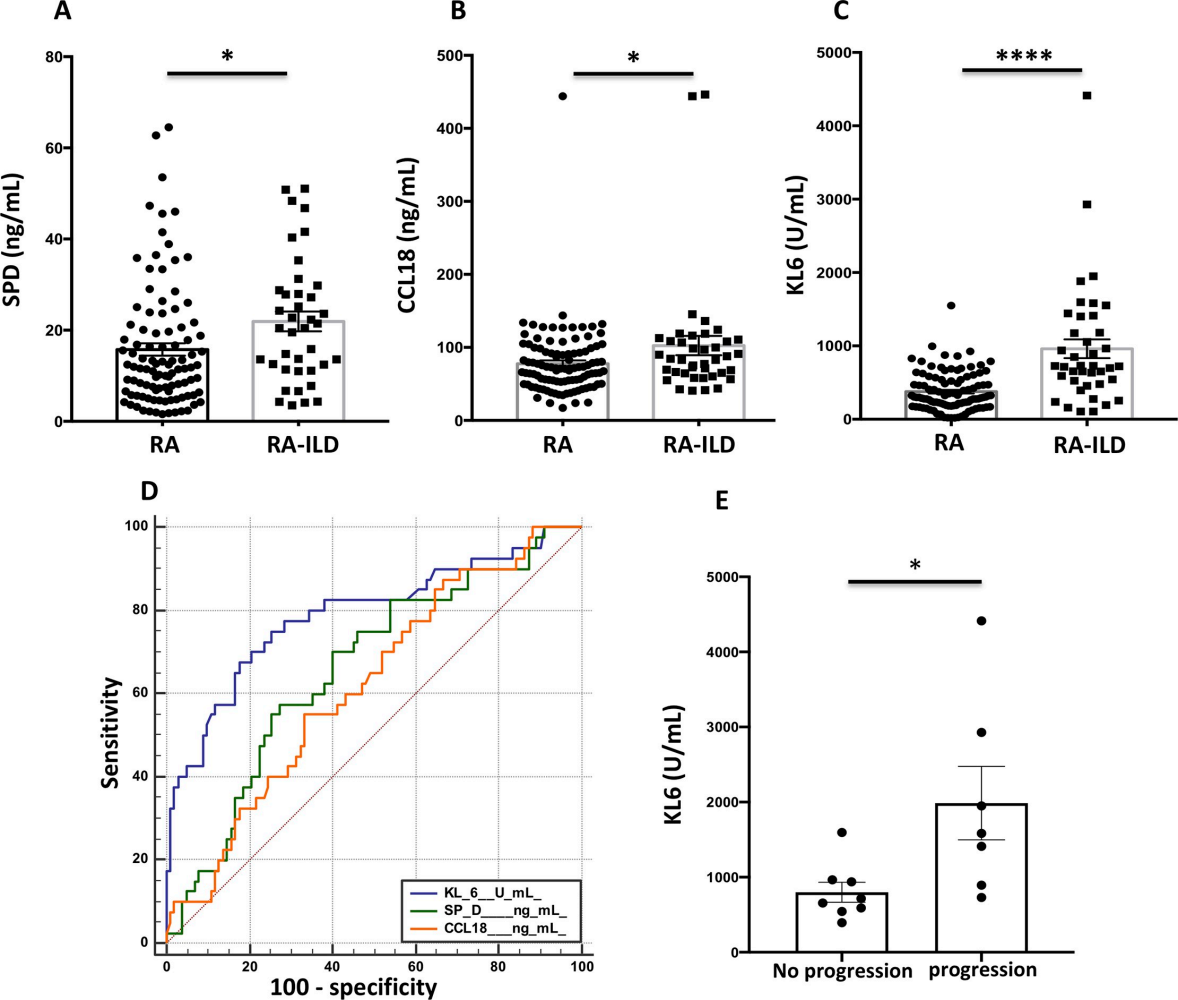
Concentrations of serum markers, diagnostic value and performance of KL-6 for the progression of rheumatoid arthritis-associated interstitial lung disease. A-C, Concentrations of SPD (ng/mL) (A), CCL18 (ng/mL) (B) and KL-6 (U/mL) (C) in patients with rheumatoid arthritis (RA) with or without associated interstitial lung disease. **D,** ROC curve illustrating the diagnostic value of SPD, CCL18 and KL-6 for diagnosis of interstitial lung disease in patients with rheumatoid arthritis. **E,** Concentrations of KL-6 (U/mL) according to the progression of rheumatoid arthritis (RA)-associated interstitial lung disease (ILD), assessed by on chest high-resolution computed tomography (HRCT) and pulmonary function tests. *p<0.05 and **** p <0.0001 by Student's t test.

SPD (21.91±2.17 *vs*. 15.76±1.34 ng/mL, p = 0.017) CCL18 (102±13 *vs*. 78±5 ng/mL, p = 0.026) and KL-6 (961±128 *vs*. 376±26 U/mL, p<0.001) concentrations (**Fig 1A–1C**) were significantly higher in patients with RA-ILD *vs*. unaffected RA patients. KL-6 values ​​were also higher in patients with UIP compared to the other HRCT patterns (980 ± 120 vs. 660 ± 95 U/mL, p = 0.040) and in patients with lesion extension> 15% (1836 ± 547 *vs*. 852 ± 120 U / mL, p = 0.031) as compared to patients with milder disease. There was no correlation between the 3 serum markers and FVC or DLCO.

ROC curve analysis to assess the diagnostic abilities of the three markers for the diagnosis of RA-ILD showed a superiority of KL-6 (Area under the curve, AUC: 0.79 95% CI 0.72–0.86, p<0.001) compared to SPD (AUC: 0.66 95% CI 0.58–0.74, p = 0.030) and CCL18 (AUC: 0.62, 95% CI 0.53–0.70, p = 0.007) (**Fig 1D**). For a threshold defined by the ROC curve at 632 U/mL, the sensitivity of KL-6 for the diagnosis of RA-ILD patients in our cohort was 68%, the specificity 83%, the PPV 60% and the NPV 87% (**Table 2**).

**Table 2:** Diagnostic performance of circulating markers, alone or in combination, for the diagnosis of RA-ILD.

	Number of patients with positive test or combination	Sensitivity	Specificity	Positive Predictive Value	Negative Predictive Value
KL-6 > 632U/mL*	**45**	**68%**	**83%**	**60%**	**87%**
SPD >19 ng/mL*	**52**	**57.5%**	**72.5%**	**45%**	**81%**
CCL18 > 83 ng/mL*	**57**	**55%**	**67%**	**39%**	**79%**
**KL-6 > 632 U/mL and SPD > 19 ng/mL**	**26**	**73%**	**91.5%**	**61.5%**	**95%**
**KL-6 > 632 U/mL and CCL18 > 83 ng/mL**	**22**	**71%**	**94%**	**68%**	**95%**
**SPD >19 ng/mL And CCL18 > 83 ng/mL**	**22**	**61%**	**93%**	**64%**	**92%**
**KL-6 > 632 U/mL and SPD > 19 ng/mL and CCL18 > 83 ng/mL**	**13**	**77%**	**97%**	**69%**	**97%**

* Optimal Thresholds of the different markers were defined by the analysis of ROC curves

The diagnostic ability of serum markers was improved when they were combined (**Table 2**). The optimal combination was the association of increased levels of KL-6 (>632 U/mL), SPD (>19 ng/mL) and CCL18 (>83 ng/mL), which increased sensitivity from 68% to 77%, specificity from 83% to 97%, PPV from 60% to 69% and NPV from 87% to 97% (**Table 2**).

We also investigated the weight of serum markers for the diagnosis of RA-ILD, in comparison with known RA-ILD risk factors including age at onset, disease duration, smoking status, gender, ACPA, CRP levels, treatment with MTX and targeted biologic therapies. In all different regression models tested, increased KL-6 levels (>632 U/mL) remained independently associated with the presence of RA-ILD (**Table 3**).

**Table 3:** Multivariate logistic regression analysis including RA-ILD as the dependent variable and RA-ILD risk factors as covariates.

Variables	Odds ratio (95% CI)	p-value
**Model 1**		
**Age at onset (years)**	1.10 (1.02–1.19)	0.015
**Disease duration**	1.16 (1.03–1.30)	0.010
**Male gender**	3.02 (0.61–15.03)	0.177
**KL-6**	72.77 (9.82–539.01)	<0.001
**Past or current smoker**	1.89 (0.35–10.18)	0.457
**ACPA levels**	1.01 (0.99–1.01)	0.094
**Methotrexate use**	0.12 (0.01–0.22)	0.001
**Targeted biologic therapy use**	1.47 (0.31–7.04)	0.630
**Model 2**		
**Age at onset (years)**	1.06 (1.01–1.12)	0.041
**Disease duration**	1.11 (1.02–1.21)	0.018
**Male gender**	1.57 (0.44–5.63)	0.487
**SPD**	6.16 (1.79–21.24)	0.004
**Past or current smoker**	1.38 (0.38–5.04)	0.629
**ACPA levels**	1.11 (0.26–4.71)	0.327
**Methotrexate use**	0.17 (0.03–0.36)	<0.001
**Targeted biologic therapy use**	2.86 (0.78–10.39)	0.111
**Model 3**		
**Age at onset (years)**	1.06 (1.00–1.12)	0.028
**Disease duration**	1.10 (1.01–1.20)	0.025
**Male gender**	1.97 (0.57–6.79)	0.279
**CCL18**	1.74 (0.58–5.24)	0.326
**Past or current smoker**	1.92 (0.58–6.34)	0.283
**ACPA levels**	0.94 (0.31–2.90)	0.299
**Methotrexate use**	0.11 (0.04–0.33)	<0.001
**Targeted biologic therapy use**	2.32 (0.72–7.49)	0.159
**Model 4**		
**Age at onset (years)**	1.10 (1.02–1.19)	0.019
**Disease duration**	1.16 (1.03–1.31)	0.013
**Male gender**	2.30 (0.41–13.02)	0.345
**KL-6**	60.37 (7.95–458.26)	<0.001
**SPD**	4.20 (0.84–21.14)	0.081
**CCL18**	1.25 (0.30–5.29)	0.760
**Past or current smoker**	1.25 (0.20–7.83)	0.808
**ACPA levels**	1.02 (0.97–1.05)	0.08
**Methotrexate use**	0.12 (0.02–0.38)	<0.001
**Targeted biologic therapy use**	2.44 (0.43–13.76)	0.313
**Model 5**		
**KL-6**	7.81 (3.02–20.17)	<0.001
**SPD**	2.80 (1.08–7.26)	0.033
**CCL18**	2.14 (0.81–5.62)	0.120
**ACPA levels**	1.14 (0.38–3.40)	0.356
**CRP levels**	1.33 (0.41–4.27)	0.633

CI: confidence Interval, ACPA: anti-citrullinated protein antibodies, CRP: C-reactive protein

### Performance of KL-6 for the progression of RA-ILD on HRCT in the French cohort

**Fig 2:**
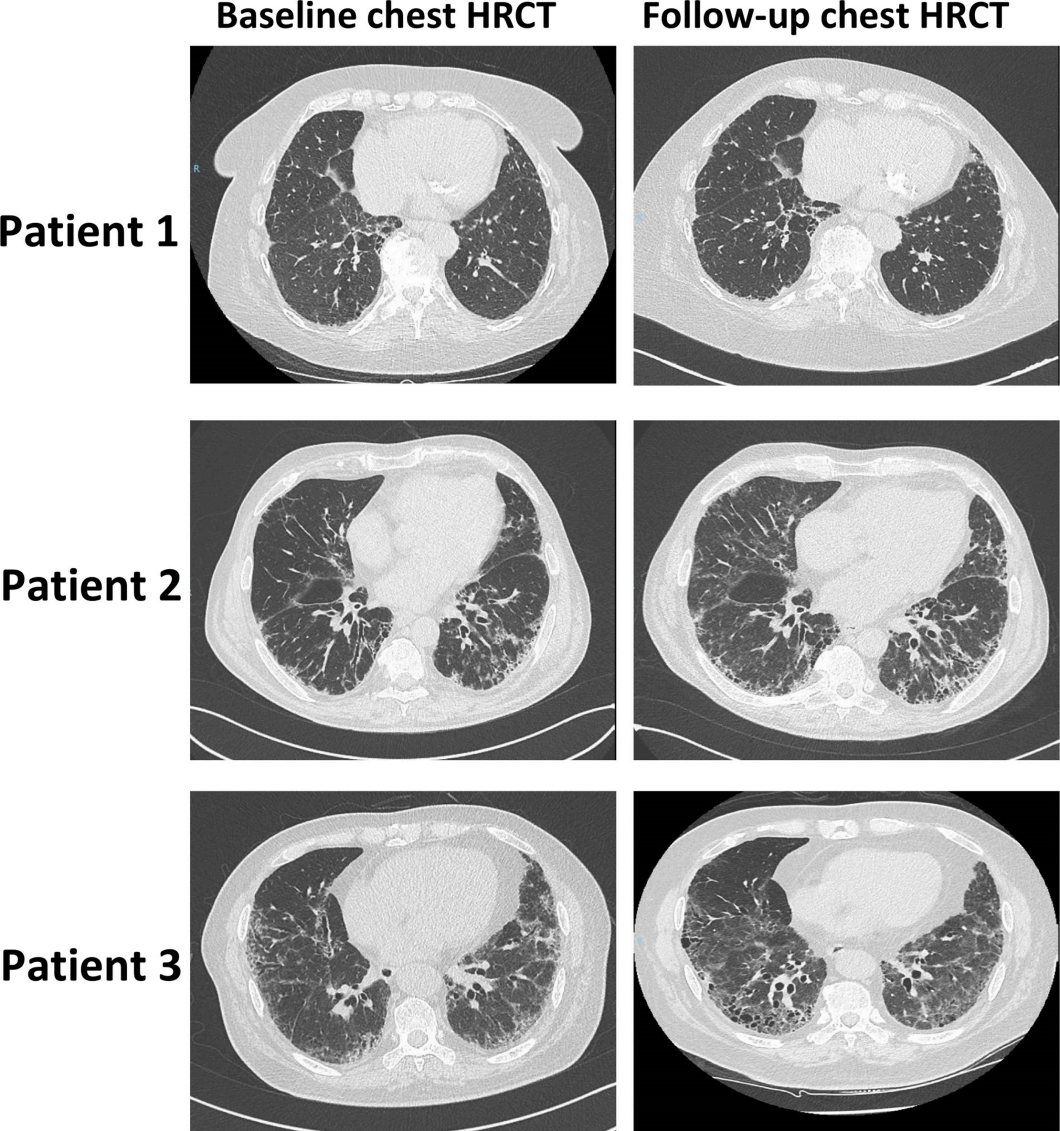
Representative baseline and follow-up chest high-resolution computed tomography performed for 3 patients with rheumatoid arthritis (RA)-associated interstitial lung disease (ILD). Patient 1: baseline KL-6 concentration of 543 U/mL, with no ILD progression detected on chest high-resolution computed tomography (HRCT). Patient 2: baseline KL-6 concentration of 1,412 U/mL, with ILD progression detected on chest HRCT. Patients 3: baseline KL-6 concentration of 4,414 U/mL, with ILD progression detected on chest HRCT.

Paired chest HRCT were available for the 15 RA-ILD French patients, with a mean time of 3±1.5 years between both examinations. RA-ILD progression was detected on 7/15 (47%) patients (**Fig 2, Table 4**). The treatment profile of RA-ILD progressors and non-progressors was similar (**Table 4**). Baseline KL-6 serum levels were significantly increased in patients who experienced ILD progression (1987±1294 vs. 799±375 U/mL, p = 0.027) (**Fig 1E**). A stability of ILD lesions and PFTS was observed in patients with baseline KL-6 <655 UI/mL (25^th^ percentile value in French patients with RA-ILD) (**Table 4**), whereas ILD progression was detected on patients with higher serum KL-6 concentrations (**Fig 1F, Table 4**). Interestingly, the degree of ILD progression on HRCT during the follow-up period was proportional to baseline KL-6 concentrations (**S1 Fig**). No relationship was observed between SPD / CCL18 serum concentrations and RA-ILD progression.

**Table 4:** Progression of RA-ILD on HRCT and PFTs according to baseline KL-6 levels in the French cohort.

KL-6	Patients	BaselineKL-6 levels (U/mL)	Baseline HRCT extension	Final HRCT extension	Baseline FVC	Final FVC	Baseline DLCO	Final DLCO	RA-ILD progression*	Treatment received
**< 655 U/mL**	**P1**	**650**	**15%**	**15%**	**58%**	**60%**	**N.A**	**N.A**	**NO**	**RTX+LEF**
	**P2**	**543**	**15%**	**15%**	**103%**	**105%**	**86%**	**90%**	**NO**	**MTX**
	**P3**	**590**	**10%**	**10%**	**76%**	**73%**	**61%**	**57%**	**NO**	**RTX + LEF**
	**P4**	**394**	**15%**	**15%**	**75%**	**77%**	**47%**	**44%**	**NO**	**MTX**
**655≤KL-6 ≤ 955 U/mL**	**P5**	**729**	**15%**	**30%**	**100%**	**90%**	**96%**	**80%**	**YES**	**RTX+MTX**
	**P6**	**716**	**5%**	**5%**	**70%**	**69%**	**68%**	**68%**	**NO**	**RTX+MTX**
	**P7**	**893**	**30%**	**40%**	**95%**	**80%**	**55%**	**44%**	**YES**	**ABA+LEF**
	**P8**	**938**	**15%**	**15%**	**75%**	**80%**	**53%**	**50%**	**NO**	**RTX + LEF**
**>955 U/mL**	**P9**	**965**	**10%**	**5%**	**71%**	**69%**	**20%**	**20%**	**NO**	**RTX+AZA**
	**P10**	**2928**	**10%**	**15%**	**105%**	**87%**	**66%**	**60%**	**YES**	**RTX+MMF**
	**P11**	**1583**	**15%**	**30%**	**79%**	**67%**	**37%**	**25%**	**YES**	**MTX**
	**P12**	**1950**	**50%**	**65%**	**123%**	**120%**	**60%**	**45%**	**YES**	**TCZ+AZA**
	**P13**	**1596**	**30%**	**30%**	**69%**	**63%**	**31%**	**33%**	**NO**	**RTX+AZA**
	**P14**	**4414**	**25%**	**35%**	**94%**	**70%**	**65%**	**55%**	**YES**	**RTX**
	**P15**	**1412**	**10%**	**15%**	**80%**	**67%**	**72%**	**65%**	**YES**	**MTX**

HRCT: high-resolution computed tomography, FVC: forced vital capacity, DLCO: diffusing capacity of lung for carbon monoxide, N.A: not available (DLCO impossible to measure for this patient), RTX: rituximab, LEF: léflunomide, MTX: methotrexate, AZA: azathioprine, MMF: mycophenolate mofetil, ABA: abatacept, TCZ: tocilizumab

*RA-ILD progression was defined by a progression >10% on HRCT and either a FVC decline >15% or a FVC decline >10% combined with a DLCO decline >15%

## Discussion

Finding reliable biomarkers for RA-ILD may promote earlier diagnosis by identifying patients at risk, improve disease stratification by predicting the subtype of RA-ILD, forecast disease course over time and guide appropriate monitoring and management. Among the 3 tested serum markers, KL-6 had the best diagnostic value for RA-ILD. However, the combination of the 3 markers improved their diagnostic ability, which might be of interest as these biomarkers reflect different compartments of lung architecture. KL-6 is a mucin-like glycoprotein, classified as MUC1 and expressed mainly on type II pneumocytes, which has profibrotic and anti-apoptotic effects on lung fibroblasts. SPD is a lipoprotein complex secreted by type II pneumocytes and Club cells to decrease surface tension at the air–liquid interface. KL-6 and SPD both reflect alveolar epithelial cells damages. CCL18 is a chemotactic factor produced by alveolar macrophages, which disclose a phenotype of alternative activation and might be a part of a positive feedback loop with lung fibroblasts perpetuating fibrotic processes [12, 13].

KL-6 has been previously identified as a serum marker for RA-ILD and more extensively for other interstitial lung diseases including idiopathic pulmonary fibrosis, which therefore may limit the scientific novelty of this research. However, one of the added value of the herein study is its sample size of RA patients for whom HRCT had been performed. Indeed, elevated KL-6 concentrations was previously suggested in a series that included 177 RA patients, but with only 9 with RA-ILD [14]. A larger study also raised some interest for KL-6 in CTD-ILD and it should be highlighted that some of these CTD-ILD had RA. This latter study showed that serum KL-6 levels were increased in CTD-ILD patients and had a positive correlation with CT grade and a negative correlation with FVC and diffusing lung for carbon monoxide (DLCO) [15].

Increased KL-6 levels were independently associated with the presence of RA-ILD after the adjustment on other known RA-ILD risk factors. This marker worked independently of antibody status and signs of inflammation, providing information beyond inflammatory parameters. This is of importance, given that issues remained on the origin of RA-ILD, which may result from various extent of inflammation or chronic fibrosing process. The MUC5B promoter variant rs35705950 was recently found associated with RA-ILD [9]. Genomics is not integrated in the standard of care of the 3 rheumatology centers for RA-ILD; thus not making it possible to include the carrier status of this MUC5B variant in our multivariate analysis.

MTX was identified as an independent protective factor of RA-ILD in all multivariate models tested. This result should be taken cautiously since it may be related to a selection bias. Indeed, RA-ILD patients were less likely to receive MTX, probably due to the theoretical risk of MTX-induced RA-ILD exacerbation. This risk is more and more debated, and recent evidence has suggested that MTX treatment is not associated with an increased risk of RA-ILD diagnosis, and, in addition, MTX may delay the onset of ILD [16].

Serum KL-6 levels were also shown to correlate with the extent of RA-ILD on computed tomography in a Japanese population of RA-ILD patients, suggesting that KL-6 may reflect the severity of the disease [17]. In our study, we show higher KL-6 concentrations in RA-ILD patients with lesion extension> 15% and in patients with the UIP pattern. In line with these results, a retrospective study performed on 84 RA-ILD patients, increased KL-6 levels were independently associated with a UIP pattern and were identified as an independent prognostic factor of mortality [18]. The degree of ILD progression on HRCT within 3 years was proportional to baseline serum KL-6 concentrations in the French subset of patients. It will be necessary to confirm these findings in prospective studies with higher sample size and examine the predictive value of KL-6 to detect worsening of pulmonary functional tests.

Although the limited sample size of our study may limit the interpretation of the results, our findings, together with available data, support that KL-6 is relevant for the diagnosis and may be interesting for the prognosis of RA-ILD. It might be used as a circulating non-invasive first-line marker to stratify for indication of HRCT. Indeed, given the emerging lung issues in RA patients, this simple and highly reproducible marker, which is already available in routine care in some countries, would be of help to risk-stratify RA patients for the performance of HRCT. Indeed, considering costs and radiation hazard, KL-6 measurement could be a good prerequisite to chest HRCT in rheumatology clinics.

**Key messages**

1/ KL-6 is a relevant marker for the diagnosis of RA-associated ILD
2/ KL-6 is helpful to risk-stratify RA patients for the performance of chest HRCT
3/ KL-6 may be predictive of RA-ILD progression

## Supporting information

S1 Fig Degree of mean ILD progression on chest high-resolution computed tomography (HRCT) according to baseline KL-6 concentrations. The concentrations of 655 UI/mL and 955 UI/mL corresponded to the first and second quartile of French patients with RA-ILD.
(PDF)
