# Supplementary material for: Improving risk-stratification of rheumatoid arthritis patients for interstitial lung disease
Source: PLoS One. 2020 May 8;15(5):e0232978. doi: 10.1371/journal.pone.0232978 (PMC7209254; doi:10.1371/journal.pone.0232978)

Figure S1: Degree of mean ILD progression on chest high-resolution computed tomography (HRCT) according to baseline KL-6 concentrations.
The concentrations of 655 UI/mL and 955 UI/mL corresponded to the first and second quartile of French patients with RA-ILD.

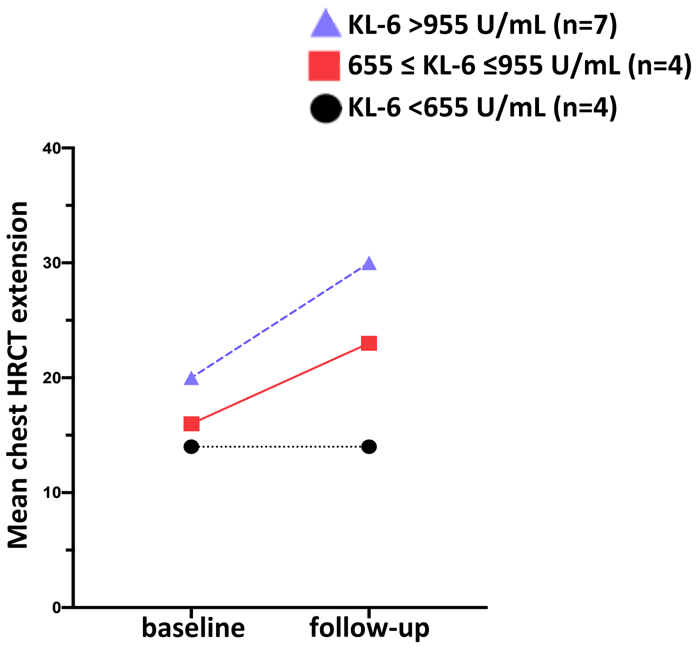

Supplement: S1 Fig — The concentrations of 655 UI/mL and 955 UI/mL corresponded to the first and second quartile of French patients with RA-ILD. (PDF) [file pone.0232978.s001.pdf]
